# Evaluating the association of depressive symptoms on serum folate and erythrocyte folate levels based on the 2017–2020 NHANES database

**DOI:** 10.3389/fnut.2025.1505700

**Published:** 2025-02-10

**Authors:** Yunhong Yang, Huaqian Qi, Jiahao Zhang, Jie Jia, Yunsong Yang, Hong Zhao

**Affiliations:** ^1^Department of Acupuncture and Moxibustion, Shanghai University of Traditional Chinese Medicine, Shenzhen Hospital, Shenzhen, China; ^2^Guangzhou University of Chinese Medicine, Guangzhou, China; ^3^The First Affiliated Hospital, Guangzhou University of Chinese Medicine, Guangzhou, China; ^4^Tianjin University of Science and Technology, Tianjin, China

**Keywords:** depressive symptoms, serum folate, erythrocyte folate, NHANES database, association

## Abstract

**Objective:**

To improve further the management of the nutritional status and dietary habits of depressed patients.

**Methods:**

This study investigated the effect of different severity states of depressive symptoms on serum and erythrocyte folate levels using the Nutrition Examination Survey (NHANES) database from 2017 to 2020. We comprised a sample of 4,872 cases from NHANES database. We developed 3 linear regression models to assess the effect of depressive symptoms on erythrocyte folate and serum folate by collating and analyzing the data. The relationship between depression severity and erythrocyte folate as well as serum folate was also mutually validated by the results of multiple logistic regression. Finally, we made restricted cubic spline plots using the glm function of R.

**Results:**

Depression remained negatively correlated with serum folate levels with a OR value of −0.02, 95% CI of −0.05 ~ −0.00. Moderate depression was negatively correlated with folate, with a OR value of −0.03, 95% CI of −0.05 ~ −0.00. When exploring the association between different degrees of depressive symptoms and erythrocyte folate, it was unexpectedly found that major depression was negatively associated with erythrocyte folate with a OR value of −0.18, 95% CI of −0.31 ~ −0.04 after adjusting for all covariates.

**Conclusion:**

Depression is associated with folate levels. The risk of serum folate insufficiency or erythrocyte folate insufficiency is higher after a positive depression. For different degrees of depressive symptoms, serum folate levels were significantly lower than normal in patients with moderate depression, while erythrocyte folate levels were lower than normal in patients with major depression. Therefore, attention should be paid to the dietary habits and nutritional status of patients with depression or depressive symptoms when they are undergoing long-term antidepressant treatment. Folic acid supplementation is recommended for patients with moderate or severe depression or for depressed patients who have developed unhealthy eating habits.

## 1 Introduction

Depression, a prevalent mental disorder, manifests through persistent low mood, cognitive slowing, impaired mental functioning, and physical symptoms. Depression is a major disorder affecting human health, with approximately 350 million people suffering from depression according to the World Health Organization, and globally it causes greater years of disability than any other disease, ranking ninth after prolific killers such as heart disease, stroke, and HIV according to a combined ranking of disability and death (1). The impact of depression extends beyond individual suffering, straining families, social functioning, and economic productivity. It also contributes significantly to suicide and premature death linked to comorbid physical conditions. Diet is a modifiable risk factor for depression, and dietary improvements can reduce the burden of depression (2). Deficiencies in essential nutrients, including folate, vitamin D, zinc, and magnesium, can disrupt brain and nervous system functions, potentially contributing to the development of depressive symptoms (3–6). Inadequate intake of these nutrients may be thus linked to worsened mental health outcomes, increasing the risk of depression. Among the essential nutrients, folate stands out due to its crucial role in various metabolic processes.

Folate is a B-group water-soluble vitamin composed of pteridine, p-aminobenzoic acid, and L-glutamic acid. Folate acts as an important cofactor mediating the transfer of one-carbon units and is involved in the metabolic processing of one-carbon units. It is involved in many biochemical pathways through its methyl transport, including neurotransmitter synthesis, DNA biosynthesis, regulation of gene expression, amino acid synthesis and metabolism, and myelin synthesis and repair (7). As a key cofactor, folate plays a central role in maintaining cellular functions across several biochemical pathways. The epidemiological and experimental evidence suggests that (8) disorders of single-carbon metabolism due to folate deficiency are associated with vascular, neurodegenerative, and neuropsychiatric diseases, among which the most prominent include cerebral ischemia, Alzheimer’s disease, and depression. Additionally, elevated homocysteine levels, a marker of folate deficiency, have been linked to cognitive decline and are considered a risk factor for cognitive impairment (9). Folic acid and vitamin B12 supplementation can reduce homocysteine levels, which may reduce the risk of cardiovascular disease and cognitive impairment (10).

A meta-analysis concluded that both serum folate levels and dietary folate intake were lower in depressed patients than in non-depressed patients, suggesting that folate supplementation may be beneficial for depressed individuals (11). Additionally, another meta-analysis indicated that folate supplementation could be suggested as an efficacious and adjuvant agent in the alleviation of depression symptoms along with routine medications (12). A further systematic review and meta-analysis supported the use of l-methylfolate as an adjunctive therapy in antidepressant treatment, particularly for adults with major depressive disorder (MDD) (13). However, some researchers have suggested contrary results, suggesting that there is no clinical benefit to using supplements such as folic acid, vitamin B6 or B12 as adjuncts to antidepressant therapy (14, 15). Whether to use folate supplementation in the management of depression in chronic illness is a controversial topic. This discrepancy may be due to the limited number of clinical studies investigating the adjunctive effects of folate or vitamin B12 supplements in depression treatment. As a result, current evidence remains insufficient, and no definitive conclusions can be drawn regarding the ability of these supplements to enhance the efficacy of antidepressant therapies (14). Folate supplementation is typically recommended only in cases of confirmed deficiency to prevent adverse outcomes. However, no studies have clearly identified a threshold value for serum folate or erythrocyte folate levels below which the risk of depression increases or when folate supplementation should be initiated. Therefore, it is crucial to utilize larger and more comprehensive datasets to systematically explore the relationship between folate levels and depression. Such analyses could help fill significant research gaps and provide evidence-based recommendations for clinical practice.

The National Health and Nutrition Examination Survey (NHANES), conducted by the National Center for Health Statistics (NCHS) and the Centers for Disease Control and Prevention (CDC), aims to assess the health and nutritional status of a representative sample of the non-institutionalized civilian population in the United States. NHANES is a highly representative national dataset that provides extensive data on biomarkers, survey responses, and physical examinations, offering valuable insights into the health and nutrition of the U.S. population. This comprehensive database enables researchers to analyze health conditions across diverse populations while controlling for various confounding factors, ensuring more generalizable and reliable conclusions. The 2017–2020 NHANES data offers detailed information on folate levels and depressive status, providing an up-to-date and representative sample for this study (16). This allows for an analysis that reflects current trends in nutrition and mental health among the U.S. population.

Based on this, this study aims to utilize the 2017–2020 NHANES database to evaluate the association between depressive status and serum folate and erythrocyte folate levels. The study seeks to address the following key questions: ① Is depression associated with lower serum and erythrocyte folate levels? ② Do these associations remain significant after adjusting for sociodemographic characteristics, lifestyle factors, and clinical confounders? ③ Is there evidence of non-linear relationships or threshold effects within these associations? Ultimately, this study aims to provide scientific evidence to improve the nutritional status and dietary habits of individuals with depression, thereby promoting more effective management of this vulnerable population and offering valuable insights for developing health management strategies for depressed patients.

## 2 Information and methods

### 2.1 Population of the study

**Figure 1:**
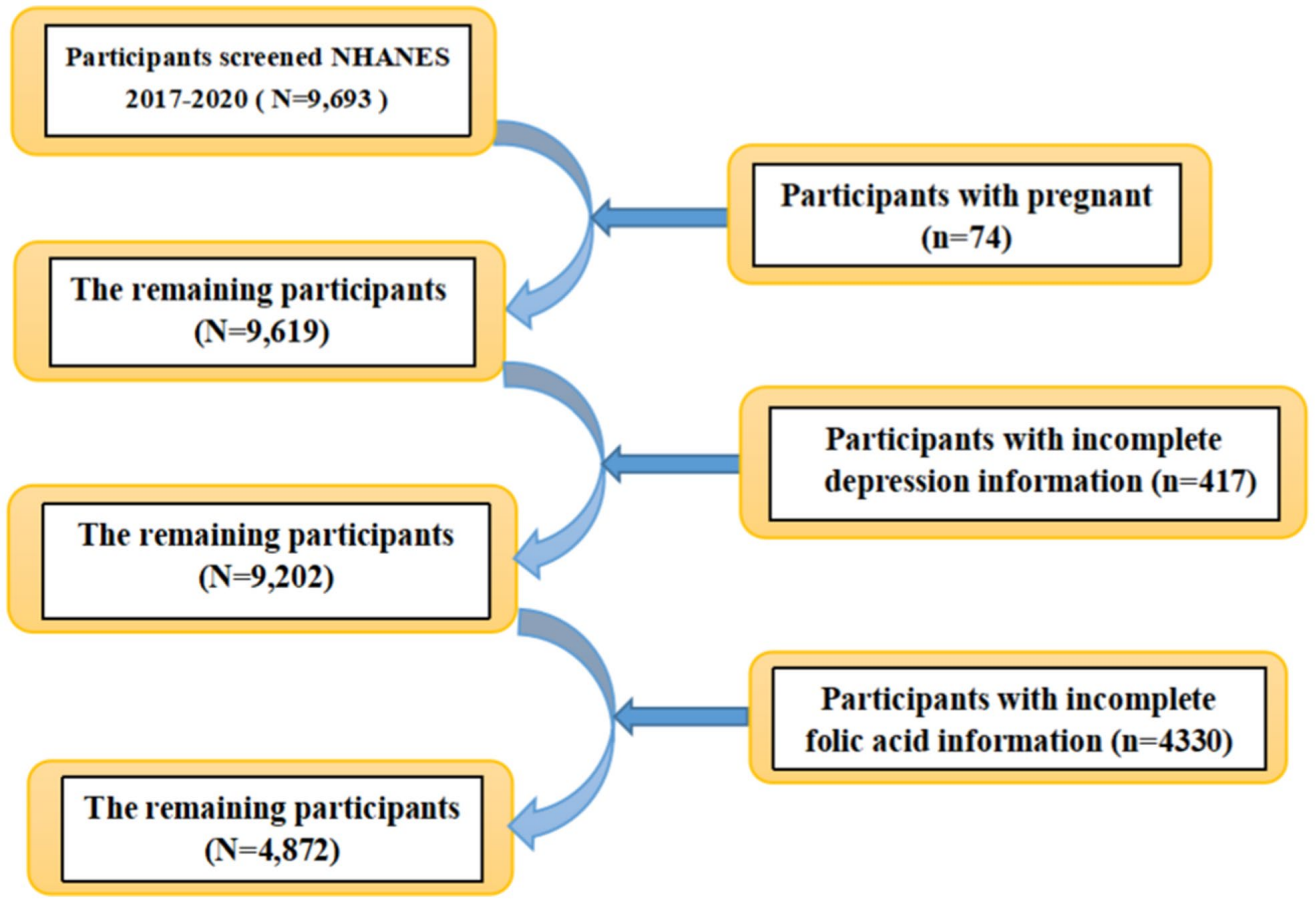
Flowchart of screening samples from NHANES.

Information from NHANES database is based on a complex multi-stage sampling design to select the sample, and the data are released every 2 years. In this study, participants were drawn from three-year consecutive US cross-sectional NHANES data from 2017 to 2020. We obtained information on 9,693 patients aged 18 years and older. We excluded female patients with laboratory test results suggestive of pregnancy (*n* = 74), as well as samples with missing information on folate measurements in laboratory tests (*n* = 4,330) and incomplete information on depression in the questionnaire (*n* = 417). Ultimately, a total of 4,872 samples were included in our study (Figure 1). We excluded pregnant patients because women are typically advised to take folic acid supplements during pregnancy to prevent fetal neural tube defects, which generally results in elevated folate levels. Additionally, hormonal changes and social factors during pregnancy can significantly impact mental health. These factors could interfere with the analysis of the relationship between folate deficiency and the risk of depression. The study was approved by the NCHS Institutional Review Board (for details see: https://www.cdc.gov/nchs/nhanes/irba98.htm) and written informed consent was obtained from all participants.

### 2.2 Determination of depression

We assessed the depressive symptoms of the participating patients using a health questionnaire (PHQ-9). Recently, the PHQ-9 has been identified as the most reliable screening tool for depression (17, 18). There were nine questions in the questionnaire and each question was scored on four levels, with each score ranging from 0 to 3, where the score represents the frequency of the symptom, e.g., 0 means not at all, 0 means the symptom occurs only for a few days, 2 means that the symptom occupies half of the respondent’s time when it occurs, and 3 means that the symptom occurs almost every day. The total score ranged from 0 to 27. The nine diagnostic items included low interest, depressed mood, sleep disturbance, poor appetite, fatigue, low self-esteem, poor concentration, slow movement or speech, suicidal ideation, and behavior. Finally, we calculated the total score of depressive symptoms. Participants with a total score of 5 and above were considered positive for depression, with 5 being the PHQ threshold (19). Total PHQ-9 scores of 5, 10, 15, and 20 represent mild, moderate, moderately severe, and severe depression, respectively (20). The questionnaire has good reliability and can effectively screen for the presence of depressive symptoms in the past 2 weeks. This has been confirmed in previous studies on depression (21, 22). The PHQ-9 has been extensively tested for depression screening and widely validated as an effective tool in primary care settings across various countries. Its psychometric reliability is well-established (23).

### 2.3 Folate measurement information

Erythrocyte folate values were obtained from the laboratory data LBDRFOSI in nmol/L and serum folate values (in nmol/L) were obtained from the laboratory data LBDFOT. The World Health Organization recommends an erythrocyte folate concentration of 906 nmol/L as the optimal concentration threshold (24) and a serum folate concentration of 15.9 nmol/L as the optimal concentration threshold (25). We designated erythrocyte folate concentrations <340 nmol/L as erythrocyte folate deficiency, erythrocyte folate concentrations between 340 and 906 nmol/L as erythrocyte folate insufficiency, and ≥906 nmol/L as adequate erythrocyte folate values. We defined serum total folate concentrations <10 nmol/L as serum folate deficiency, concentrations between 10 and 15.9 nmol/L as serum folate insufficiency, and concentrations ≥15.9 nmol/L as normal category.

### 2.4 Covariates

Based on previous literature and available information in the NHANES database, a number of confounding factors were included in the current study: ① Demographic and socioeconomic status: including age, BMI, gender, race/ethnicity (Mexican American, Other Hispanic, Non-Hispanic White, Non-Hispanic Black, Non-Hispanic Asian or Other Race), educational attainment (≤High school, >High school or Missing), Poverty Income Ratio (PIR ≤ 2.0, >2.0 or Missing), Marital Status (Married/Living with Partner, Non-married/Widowed/Divorced/Separated, Missing). ② Previous poor lifestyle habits: including whether there is a history of alcohol consumption, smoked at least 100 cigarettes in life, vigorous work activity, moderate work activity, moderate recreation, usually work 35 or more hours per week, and how healthy is the diet. ③ Previous health conditions (whether suffering from chronic diseases, etc.): including cardiovascular health conditions (Grade 1 Angina, Grade 2 Angina, Normal or Missing), whether with hypercholesterolemia, diabetes mellitus, hypertension and hysterectomy (Table 1). These covariates were selected to ensure a more accurate analysis and minimize bias in understanding the relationship between folate and depression.

**Table 1:** General demographic characteristics.

	RBC folate				Serum total folate			
	Deficiency	Insufficiency	Normal	*P*-value^a^	Deficiency	Insufficiency	Normal	*P*-value
Age (years)	46.12 ± 13.26	39.95 ± 15.85	47.21 ± 17.39	<0.0001	43.48 ± 16.57	41.13 ± 14.88	45.33 ± 17.41	<0.0001
BMI^b^	29.50 ± 8.92	28.90 ± 7.52	30.12 ± 7.27	<0.0001	34.16 ± 7.76	31.93 ± 8.84	29.50 ± 7.18	<0.0001
Gender				0.0046				0.3241
Male	46.15	35.67	40.62		30.47	40.88	39.07	
Female	53.85	64.33	59.38		69.53	59.12	60.93	
Race				<0.0001				<0.0001
Mexican American	51.32	10.47	8.86		7.64	8.35	9.44	
Other Hispanic	35.4	7.53	7.46		3.15	5.57	7.68	
Non-Hispanic White	13.28	52.87	65.87		49.94	54.8	62.62	
Non-Hispanic Black		17.55	8.43		34.6	18.64	10.34	
Non-Hispanic Asian	51.32	5.99	5.24		0.94	5.28	5.54	
Other Race	35.4	5.59	4.15		3.74	7.35	4.38	
Education level				0.0001				0.0001
≤High school	53.83	39.01	33.32		56.09	42.52	34.2	
>High school	43.9	56.56	63.5		43.91	54.97	62.1	
Missing	2.27	4.43	3.18			2.51	3.69	
Marital status				0.0003				0.0377
Married/Living with Partner	44.79	55.05	61.66		45.11	59.67	59.79	
Non-married/Widowed/Divorced/Separated	52.94	40.49	35.17		54.89	37.66	36.53	
Missing	2.27	4.46	3.17			2.67	3.68	
PIR^c^				<0.0001				0.0001
≤2	78.38	33.35	26.83		45.64	37.52	27.97	
>2	8.94	55.77	62.91		48.36	52.92	61.45	
Missing	12.67	10.88	10.25		6	9.56	10.58	
Alcohol	97.4	93.89	92.26	0.1684	95.36	96.43	92.42	0.0638
Smoked at least 100 cigarettes in life	77.37	42.23	38.75	0.0079	63.54	54.39	38.35	<0.001
Vigorous work activity	55.14	28.63	25.73	0.0815	23.39	31.44	26.3	0.2748
Moderate work activity	45.8	47.47	49.87	0.3179	38.06	46.72	49.47	0.2248
Moderate recreation	9.28	42.63	51.06	<0.0001	25.95	40.41	49.38	0.0001
Usually work 35 or more hours per week	7.68	6.88	7.38	0.8068	9.96	5.19	7.36	0.2793
How healthy is the diet				<0.0001				<0.0001
Excellent	6.34	5.36	6.74		0.51	3.1	6.65	
Very good	53.67	16.73	22.71		9.05	10.09	21.92	
Good	39.99	40.53	40.52		32.57	40.25	40.67	
Fair		29.58	24.45		43.95	35.17	25.04	
Poor	6.34	7.77	5.58		13.91	11.39	5.71	
Missing	53.67	0.03	0.00		0.00	0.00	0.01	
Coronavirus disease				<0.0001				0.0002
Grade 1 Angina	3.74	0.92	1.75		0.7	0.91	1.56	
Grade 2 Angina	25.64	0.41	0.67		3.46	1.32	0.53	
Normal	44.5	44.19	58.74		52.01	45.01	55.08	
Missing	26.12	54.48	38.84		43.82	52.76	42.82	
Diabetes	28.24	4.86	11.5	<0.001	10.84	8.85	9.54	<0.0001
Hypertension	64.36	23.12	31.71	<0.001	46.18	29.78	28.86	0.0731
Hypercholesterolemia	100	22.89	33.72	<0.001	23.64	25.58	30.84	0.025
Hysterectomy	51.58	7.14	10.26	<0.0001	6.15	9.53	9.31	0.3177

^aP < 0.05 was considered statistically significant.^

^b^Body Mass Index (kg/m^2^).

^cRatio of family income to poverty.^

The inclusion of covariates based on demographic, socioeconomic, lifestyle, and health status variables is essential for controlling potential confounding factors, thereby improving the accuracy and reliability of the study’s results (26). Demographic characteristics such as age, gender, race, and socioeconomic status, as well as lifestyle and health conditions, are generally considered important covariates (27). Additionally, previous research has shown that chronic diseases such as diabetes, hypertension, and cardiovascular conditions are closely associated with depression (28, 29). These conditions or their treatments may influence the metabolism or utilization of folate, thus affecting the occurrence of depression. Based on prior findings from our team, hysterectomy has also been found to impact the onset of depression (20). Therefore, controlling for these factors is crucial for improving the accuracy of the study’s results.

### 2.5 Statistical methods

All analyses in this study were performed using EmpowerStats statistical software (X&Y Solutions, Boston, MA) and R software (version 3.4.3), and *p* < 0.05 was considered a significant difference. We used sample weights to appropriately estimate data representing the deinstitutionalized civilian population of the United States, considering the intricate design of NHANES. The data are reported as mean ± SD and Min–Max for continuous variables and percentages for categorical variables (*N*%). And *p*-values for continuous and categorical variables were calculated using linear regression models and chi-square tests. To explore the effects of depressive symptoms on erythrocyte folate as well as serum folate, we developed 3 linear regression models. Model 1 represents unadjusted outcomes. Model 2 corrected for age, sex, race, body mass index, education, marital status and poverty income status. And model 3 corrected for all covariates listed in Table 1. In addition, the relationship between depression severity and erythrocyte folate as well as serum folate was mutually validated by the results of multiple logistic regression. Finally, The glm function of the R language was used to do fit the generalized linear model, and to maintain the smoothness and stability of the curve, restricted cubic spline plots were produced in this study. RCS plots were used to explore the non-linear relationship between serum folate levels and depression risk. Unlike linear models, RCS allows for more flexibility in capturing complex patterns, helping us better understand how serum folate changes are associated with depression risk. This method enhances the analysis of the dose–response relationship between folate levels and depression.

## 3 Results

### 3.1 Analysis of the number of participants

All relevant information of a total of 4,872 participants was entered into the analysis of the results.

### 3.2 Basic characteristics of participants

There were 4,872 participants aged 18 years or older, of whom 10 had erythrocyte folate deficiency, 1,664 had erythrocyte folate insufficiency, and 3,198 had adequate erythrocyte folate values. According to the distribution of serum folate concentrations, then there were 71 participants with serum folate deficiency, 405 with serum folate insufficiency, and 4,396 with adequate serum folate category. The covariate *p* < 0.05 was statistically significant (Table 1).

### 3.3 Results of multiple regression analysis

#### 3.3.1 Association between depressive symptoms and serum folate

As shown in Table 2, there was an association between depressive symptoms and serum folate concentrations. Even with our model 3 (i.e., adjusted for all covariates), there was still a negative association between depressive symptoms and serum folate levels, i.e., depressed patients had lower folate levels than non-depressed individuals, with a OR value of −0.02, 95% CI of −0.05 to −0.00, and a statistically significant *p*-value of 0.0383. To further explore the correlation between the two, we classified depressive symptoms and found that moderate depression was negatively associated with folate with a OR value of −0.03, 95% CI of −0.05 to −0.00, and a *p*-value of 0.0477, which showed a statistical difference. These findings suggest that lower serum folate levels may be linked to the presence of depressive symptoms, particularly in those with moderate depression, highlighting the potential role of folate in depression.

**Table 2:** Multivariate regression analysis of the association between depressive symptoms and serum folate concentration.

Exposure		Non-adjusted			Adjust I			Adjust II		
		OR	(95%CI)	*P*	OR	(95%CI)	*P*	OR	(95%CI)	*P*
Positive for depression	No		–			–			–	
	Yes	−0.05	(−0.07, −0.02)	<0.0001	−0.04	(−0.06, −0.02)	0.0004	−0.02	(−0.05, −0.00)	0.0383
Severity of depression	A		–			–			–	
	B	−0.05	(−0.07, −0.02)	0.0008	−0.04	(−0.07, −0.01)	0.0023	−0.03	(−0.05, −0.00)	0.0477
	C	−0.06	(−0.10, −0.01)	0.0083	−0.05	(−0.09, −0.01)	0.0259	−0.03	(−0.07, 0.01)	0.2089
	D	−0.01	(−0.07, 0.06)	0.8145	−0.01	(−0.07, 0.06)	0.8703	0.02	(−0.04, 0.09)	0.5144
	E	−0.10	(−0.20, 0.01)	0.0712	−0.09	(−0.19, 0.01)	0.0891	−0.07	(−0.17, 0.03)	0.1732

Positive for depression: Participants with an overall score of 5 and above are considered to be positive for depression, with 5 being the PHQ threshold.

Non-adjusted: It refers to model 1, without adjusting for any covariates.

Adjust Ι: It means model 2, adjusted for covariates including gender, age, race, BMI, marital status, and educational attainment.

Adjust Π: It refers to model 3, adjusting for all covariates.

A: No depression.

B: Mild depression.

C: Moderate depression.

D: Moderate to severe depression.

E: Major depression.

#### 3.3.2 Relationship between depressive symptoms and erythrocyte folate

We found that when exploring the association between depression and erythrocyte folate, Model 1 had a OR value of −0.03, with a 95% CI of −0.06 ~ −0.00. The results were consistent for Models 2 and 3, with a OR value of −0.02 with a 95% CI of −0.05 ~ 0.01. However, when exploring the relationship between different degrees of depressive symptoms and erythrocyte folate, it was unexpectedly found that major depression was negatively correlated with erythrocyte folate with OR values of −0.18 with 95% CI of −0.32 to −0.05, −0.32 to −0.04 and − 0.31 to −0.04 for unadjusted covariates, adjusted for some covariates and adjusted for all covariates, respectively, *p*-values were 0.0094, 0.0091 and 0.0097 (Table 3). These results suggest a significant negative association between major depression and erythrocyte folate levels, with the relationship remaining consistent even after adjusting for covariates.

**Table 3:** Multivariate regression analysis of the association between depressive symptoms and RBC folate concentration.

Exposure		Non-adjusted			Adjust I			Adjust II		
		OR	(95%CI)	*P*	OR	(95%CI)	*P*	OR	(95%CI)	*P*
Positive for depression	No		–			–			–	
	Yes	−0.03	(−0.06, −0.00)	0.0357	−0.02	(−0.05, 0.01)	0.1437	−0.02	(−0.05, 0.01)	0.3228
Severity of depression	A		0			–			–	
	B	−0.02	(−0.06, 0.01)	0.179	−0.02	(−0.05, 0.02)	0.3872	−0.01	(−0.04, 0.03)	0.6279
	C	−0.03	(−0.09, 0.02)	0.2433	−0.02	(−0.07, 0.03)	0.4733	−0.01	(−0.07, 0.04)	0.6309
	D	−0.03	(−0.11, 0.06)	0.5464	−0.02	(−0.10, 0.07)	0.7207	−0.01	(−0.09, 0.08)	0.9067
	E	−0.18	(−0.32, −0.05)	0.0094	0.18	(−0.32, −0.04)	0.0091	−0.18	(−0.31, −0.04)	0.0097

Positive for depression: Participants with an overall score of 5 and above are considered to be positive for depression, with 5 being the PHQ threshold.

Non-adjusted: It refers to model 1, without adjusting for any covariates.

Adjust Ι: It means model 2, adjusted for covariates including gender, age, race, BMI, marital status, and educational attainment.

Adjust Π: It refers to model 3, adjusting for all covariates.

A: No depression.

B: Mild depression.

C: Moderate depression.

D: Moderate to severe depression.

E: Major depression.

### 3.4 Analyzing the relationship between depression and serum folate using restricted cubic spline plots

#### 3.4.1 Relationship between depression scores and the incidence of serum folate insufficiency

**Figure 2:**
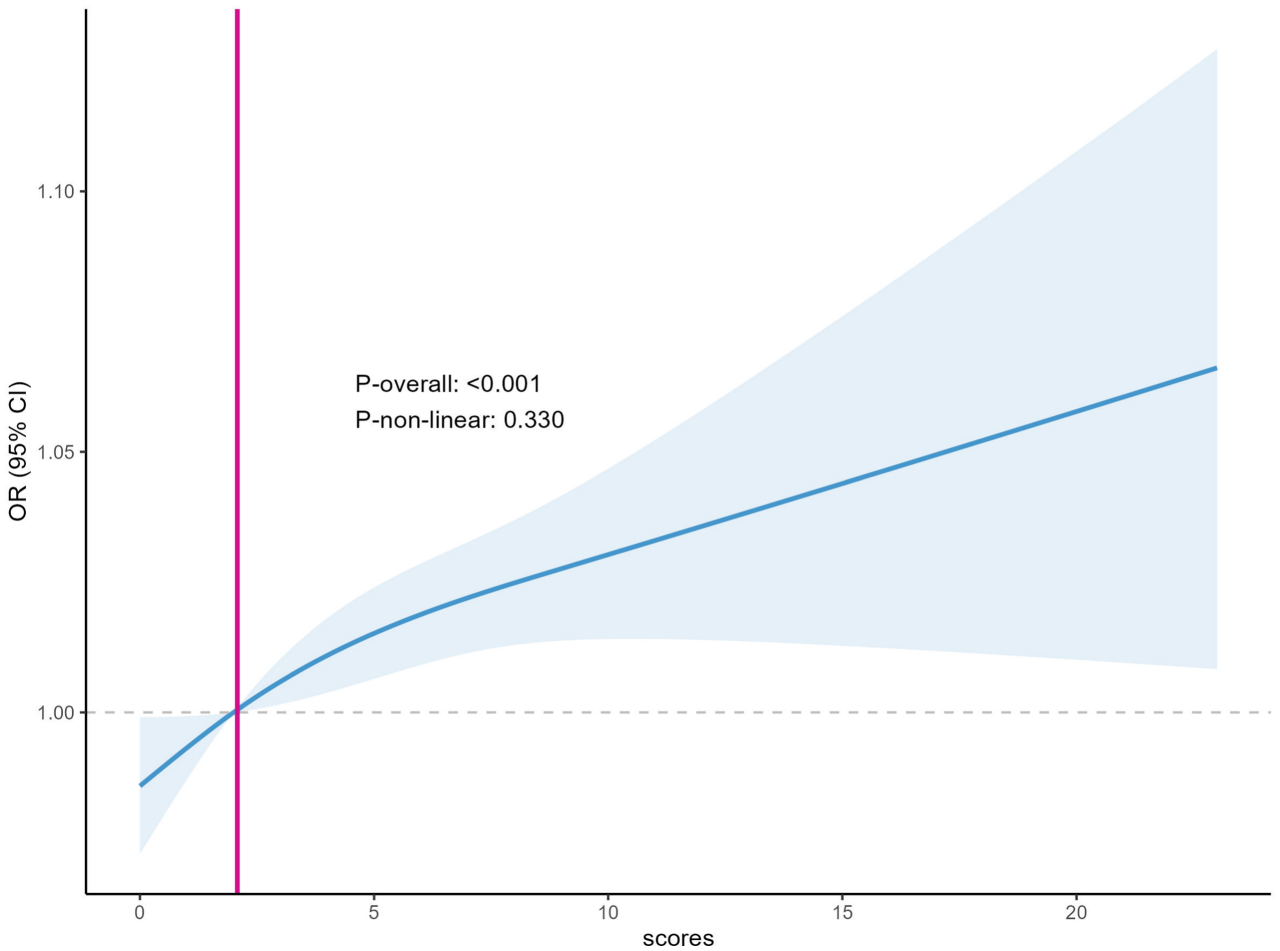
Plot of depression score in relation to the incidence of serum folate deficiency.

The results of the RCS curves showed a linear relationship between depression score and the risk of incidence of serum folate insufficiency. Specifically, the P-overall <0.001, which strongly suggests that the overall relationship between depression score and the incidence of serum folate insufficiency was highly significant. This indicates that as the depression score increases, the risk of developing serum folate insufficiency also rises. Notably, all ORs were greater than 1 after a depression score of 5 (Figure 2). The RCS plots offer a visual depiction of this linear association. On the *x*-axis, the depression scores are plotted, while the *y*-axis represents the OR of serum folate insufficiency along with its 95% CI, which is indicated by the shaded region. The blue curve, representing the fitted line, exhibits a consistent upward trajectory as the depression scores increase, thereby validating the positive linear relationship. The *P*-overall value provides robust statistical evidence supporting the significance of this overall trend.

#### 3.4.2 Relationship between serum folate levels and depression

**Figure 3:**
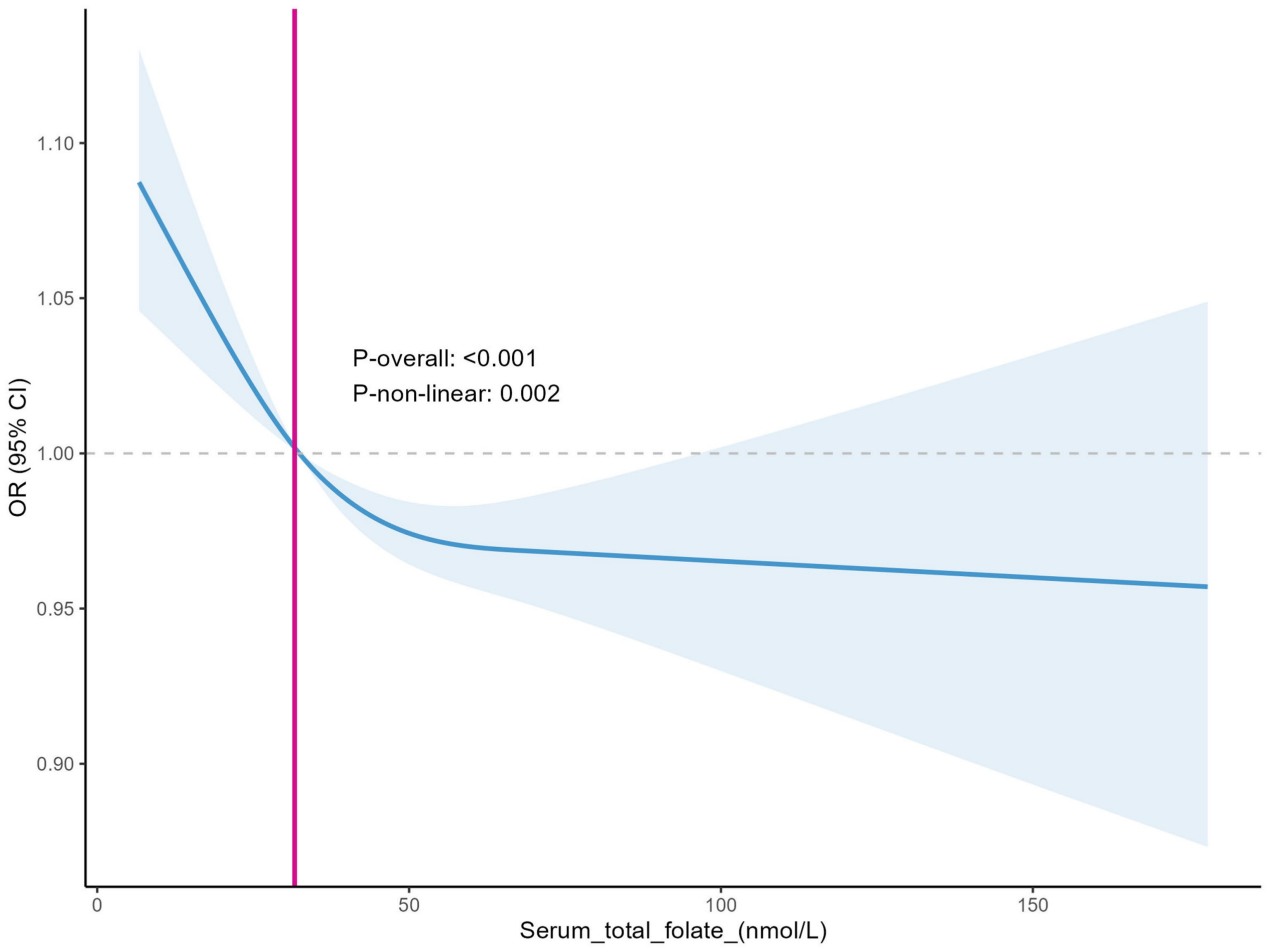
Relationship between serum folate levels and depression (Restricted Cubic Spline).

The RCS curves showed a non-linear relationship between serum folate levels and with the prevalence of depression, *P*-overall <0.001 and *P*-non-linear = 0.002. This indicates a significant non-linear trend in the relationship. In the Figure 3, the abscissa represents the serum folate level (nmol/L), and the ordinate represents the OR of depression and its 95% CI, indicated by the shaded area. When the serum folate level reaches the critical point of 31.656 nmol/L, the OR value for depression is 1. Below this threshold, as the folate level gradually decreases, the OR value shows an upward trend, which means that the higher the degree of folate deficiency, the greater the risk of depression, strongly suggesting that folate deficiency may be an important factor leading to an increased risk of depression. However, once serum folate levels exceed this critical point, the risk of depression begins to decline and remains relatively stable at higher folate levels (Figure 3), which implies that there is a specific range of serum folate levels within which the impact on the risk of depression is more significant, and the impact is relatively weakened beyond this range.

**Figure 4:**
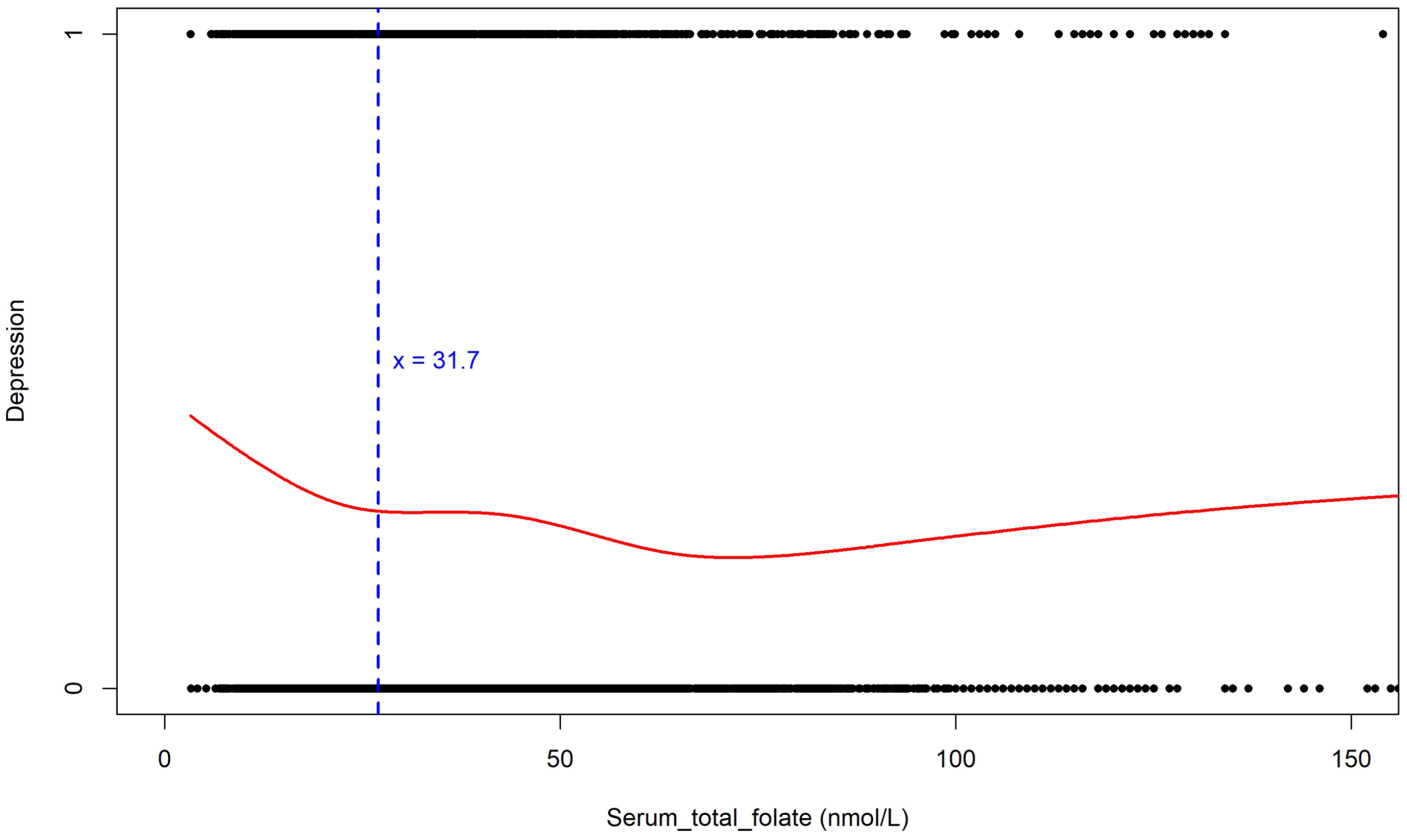
Relationship between serum folate levels and depression (Generalized Additive Models).

To further explore the potential non-linear relationship and possible threshold effects, we conducted supplementary analyses using Generalized Additive Models (GAM). The analysis results are highly consistent with the previous analysis based on the RCS curves, once again confirming that when the serum total folate level is below approximately 31.7 nmol/L, the risk of depression will significantly increase (Figure 4). In the figure, the red curve shows the Depression – Serum_total_folate relationship. The blue dashed line at *x* = 31.7 is a key threshold. Below it, the curve descends, meaning lower folate levels correlate with higher Depression levels, as in the RCS curves. Above it, the curve fluctuates but stays relatively low, indicating that beyond this point, the risk of depression drops and stabilizes at higher folate levels. The black dots are data points, supporting the trend. This GAM result validates the non-linear relationship and threshold effect, strengthening the study’s conclusions.

### 3.5 Analyzing the relationship between depression and erythrocyte folate using restricted cubic spline plots

#### 3.5.1 Relationship between depression scores and the incidence of erythrocyte folate insufficiency

**Figure 5:**
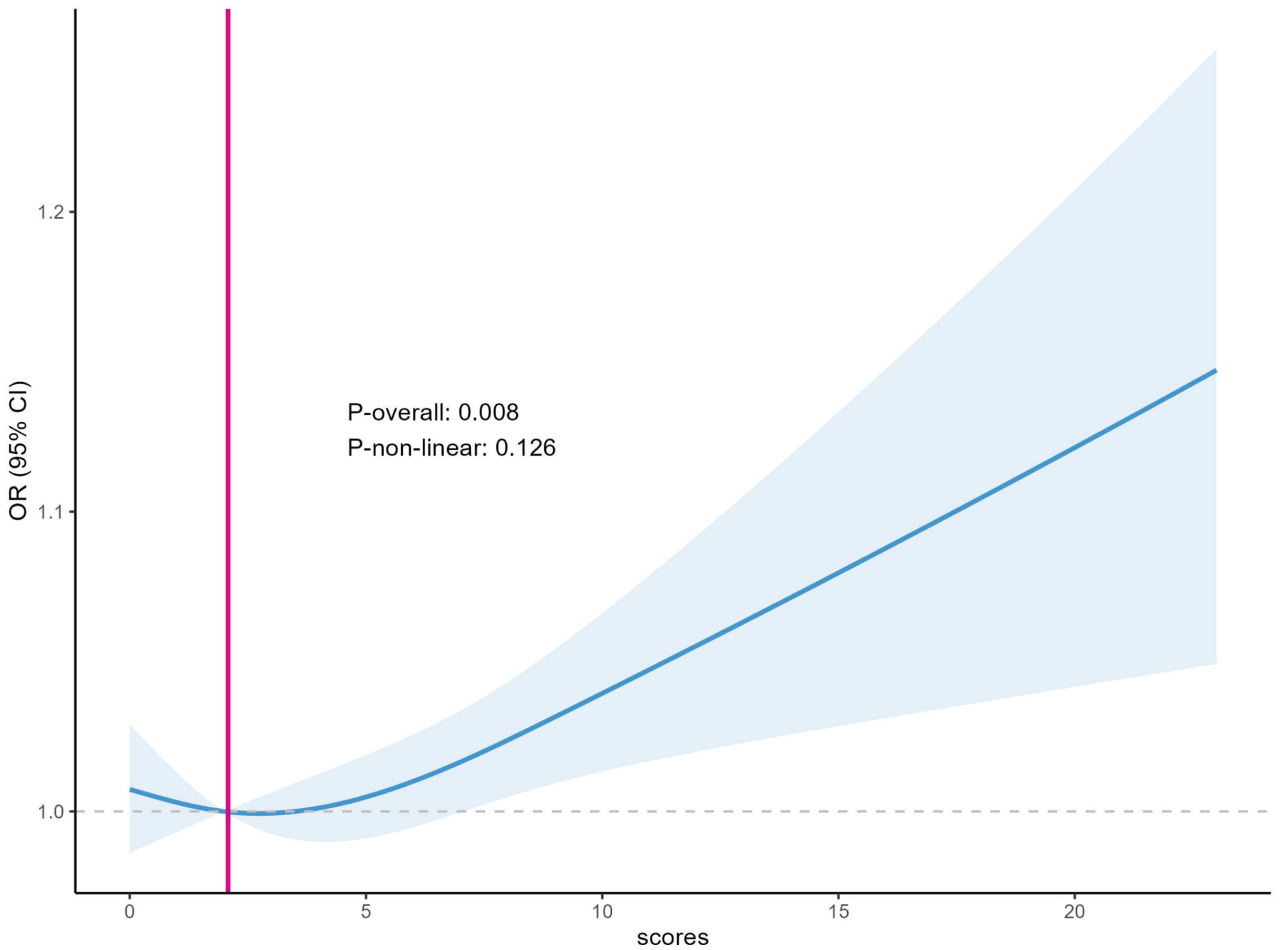
Plot of depression score in relation to the incidence of erythrocyte folate deficiency.

The RCS curve showed a linear relationship between depression score and the risk of developing erythrocyte folate insufficiency, *p*-overall = 0.008. This indicates that the relationship between depression score and the incidence of erythrocyte folate insufficiency is significant overall. Specifically, an increase in depression score was significantly associated with an increased risk of erythrocyte folate insufficiency, as indicated by an elevated odds ratio, a finding that was statistically significant (Figure 5). The RCS plots visually present this linear association. On the x-axis, the depression scores are plotted, while the y-axis represents the OR of erythrocyte folate insufficiency along with its 95% CI, which is indicated by the shaded region. The blue curve, symbolizing the fitted line, displays a consistent upward trend as the depression scores increase, thereby confirming the positive linear relationship.

#### 3.5.2 Relationship between erythrocyte folate levels and depression

**Figure 6:**
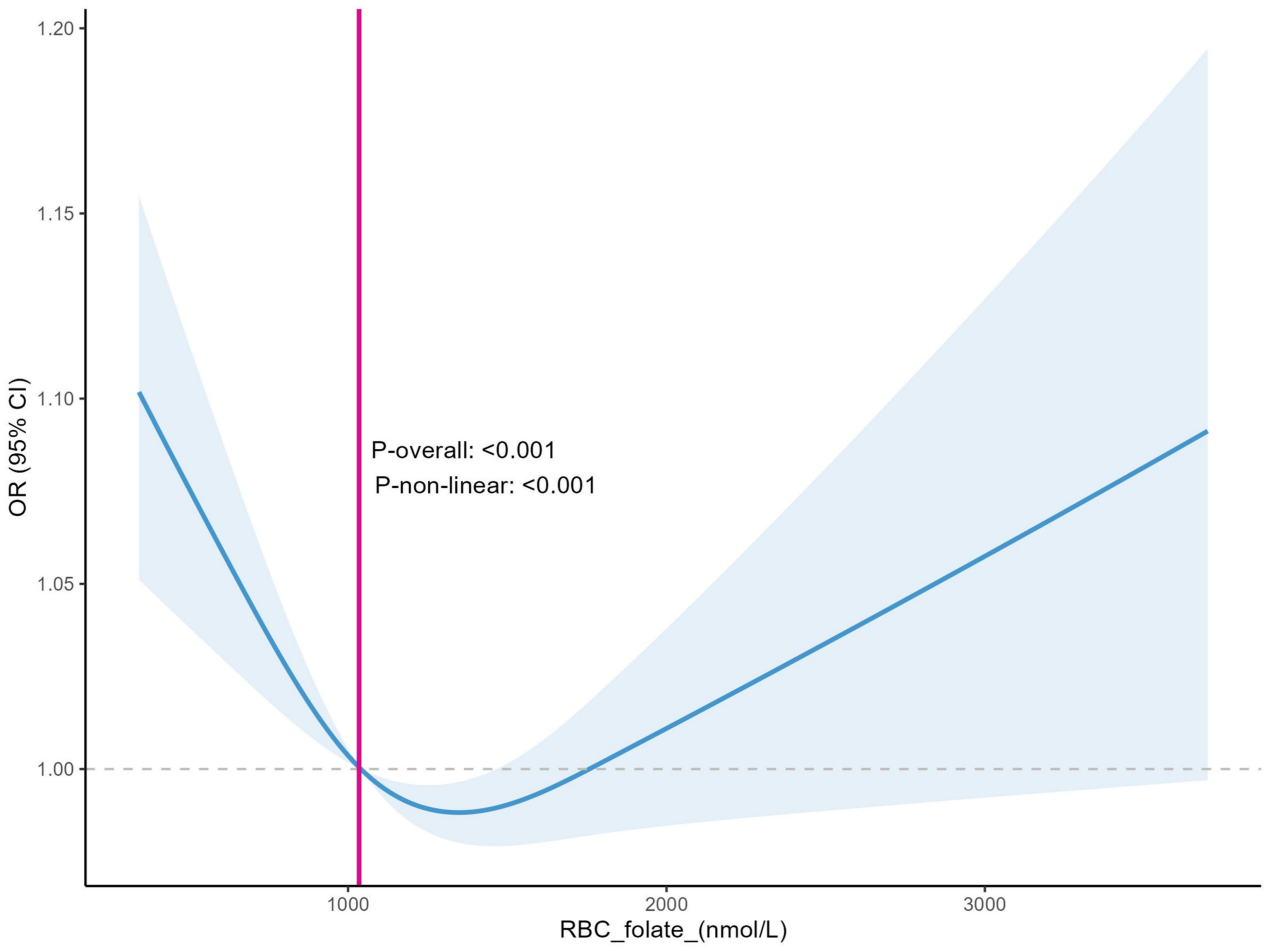
Relationship between erythrocyte folate levels and depression (Restricted Cubic Spline).

The RCS curves demonstrated a non-linear relationship between erythrocyte folate levels and with the prevalence of depression, with *P*-non-linear <0.001. This indicates a significant non-linear trend in the relationship. In Figure 6, the abscissa represents the erythrocyte folate level (nmol/L), and the ordinate represents the OR of depression and its 95% CI, indicated by the shaded area. When the erythrocyte folate level reaches approximately 1034.6 nmol/L, the OR value for depression starts to decrease as the folate concentration further drops below this point, suggesting that lower erythrocyte folate levels within this range may be associated with a reduced risk of depression. On the other hand, when the erythrocyte folate levels exceed around 1760 nmol/L, the OR for depression begins to increase, indicating that higher erythrocyte folate levels beyond this threshold may be linked to an elevated risk of depression (Figure 6). This implies that there are specific ranges of erythrocyte folate levels within which the impact on the risk of depression varies significantly.

**Figure 7:**
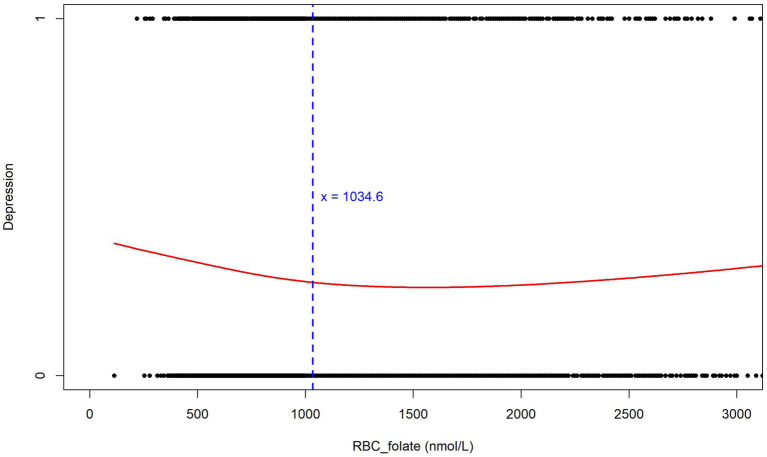
Relationship between erythrocyte folate levels and depression (Generalized Additive Models).

However, to further explore the non-linear relationship and possible threshold effects, we performed an analysis using GAM. The analysis results are highly consistent with the previous analysis based on the RCS curves. In Figure 7, the red curve depicts the relationship between Depression and RBC_folate (nmol/L). The blue dashed line at *x* = 1034.6 serves as a crucial threshold. Below this value, the curve shows a downward trend, indicating that when erythrocyte folate levels are low (below 1034.6 nmol/L), the risk of depression is higher, which aligns with the observation from the RCS curves. However, as erythrocyte folate levels increase beyond this point, the curve exhibits a slight upward trend, suggesting that higher erythrocyte folate levels may also be associated with an elevated risk of depression. The black dots scattered along the graph represent individual data points, reinforcing the overall trend observed in the curve (Figure 7). This GAM result validates the non-linear relationship and threshold effect, thereby strengthening the conclusions of the study.

## 4 Discussion

Depression is a common mental disorder, with more than 15% of the US population suffering from it (30). In the last decade, the rate of depression among young people has increased alarmingly, and the adverse outcomes associated with depression include recurrence of depression; episodes of other psychiatric disorders, and long-term impairment in interpersonal, social, and occupational functioning (31). In the 1970s, the term major depressive disorder (MDD) began to appear in the United States, and 10 years later MDD was formally incorporated into the Diagnostic and Statistical Manual of Mental Disorders-III (DSM) (32). Surveys have found that patients with major depressive disorder experience one or more depressive episodes during their lifetime, with an estimated lifetime prevalence of 16.6% and a relapse rate of 35 to 80% within 1 year of remission (33). Suicide rates are generally elevated in MDD especially among younger patients, with at least 10% experiencing suicidal ideation and suicidal behavior (34), and its social impact is considerable.

Folate is widely available in foods of plant and animal origin especially rich in fresh green vegetables and fruits. Some foods and dietary habits are known to affect mental health, and Gülseren found lower severity of depression in individuals with high intakes of grains and vegetables (35). However, psychological, physiological, genetic and social functioning can influence a person’s eating behavior, food intake, and food preferences. This study found an association between depression and serum folate levels, indicating that individuals with depression have lower serum folate levels compared to those without depression. Comparisons based on the severity of depression revealed that patients with moderate depression had notably lower serum folate levels, and the *p*-values were statistically significant. These results are consistent with previous studies (11, 36), as this systematic review, which analyzed 43 studies, found that individuals with depression have significantly lower serum folate levels and dietary folate intake compared to those without depression (Hedge’s *g* = −0.24, [95% CI = −0.31, −0.16], *p* < 0.001). This may be due to the fact that depressed patients are prone to food preferences including fast food, snacks, and low-quality foods, and unhealthy eating habits in depressed patients result in reduced intake of vegetables, fruits, fish, chicken, milk, and grains (37). Previous studies have shown that dietary patterns and depression are interconnected through the mediating effects of folate and vitamin B12. The interactions between different foods in unhealthy dietary patterns can also undermine the protective effects of folate and vitamin B12 against depression (38). That is why depressed patients will be found to have lower levels of serum folate than normal in laboratory tests.

Serum folate levels can reflect recent dietary folate intake, while erythrocyte folate levels can reflect hepatic folate stores. This study unexpectedly found that major depression was negatively associated with erythrocyte folate when carefully distinguishing between different levels. However, after adjusting for covariates, some of the data on erythrocyte folate were not statistically significantly different. The discrepancy in the results of the relationship between erythrocyte folate, serum folate and depression in the present experiment may be due to the fact that erythrocyte and plasma folate concentrations, although highly correlated, are not the same biomarkers of folate status and their relationship is influenced by BMI, methylenetetrahydrofolate reductase (MTHFR) genotype and vitamin B-12 status (24). On the other hand, erythrocyte folate is thought to have a cycle turn similar to the erythrocyte lifespan (approximately equal to 120 days). This is because erythrocytes add folate only during erythropoiesis and release the vitamin during cell lysis (39). Erythrocyte folate is a marker of long-term folate status and its initial increase depends on the dose of folate or folate supplements and baseline erythrocyte folate concentration (39). However, as the prevalence of eating disorders is four times higher in those suffering from major depression than in normal subjects (40), patients with chronic depression (especially in those with major depression) may contribute to causing erythrocyte folate to be at low levels.

To better determine if erythrocyte folate and serum folate are directly related to depression? We generated restricted cubic spline plots for prediction and found that when the depression score reached ≥5 (indicating positive depression), higher depression scores were associated with an increased risk of both serum folate insufficiency and erythrocyte folate insufficiency. This is consistent with previous studies (11). However, previous studies have primarily focused on the presence of folate deficiency in patients with depression or the potential benefits of folate supplementation in improving depression (41–43). They have not specifically addressed the threshold levels of folate or erythrocyte folate below which depression may be influenced. This raises a question: Could insufficient folate or erythrocyte folate levels contribute to the development of depression? At what threshold does a deficiency in folate or erythrocyte folate increase the risk of depression? We used the GAM model to identify a non-linear relationship between both serum folate and erythrocyte folate levels and depression. The study results indicate that the risk of depression increases when serum folate levels are below 31.7 nmol/L and erythrocyte folate levels are below 1034.6 nmol/L. These values are both higher than the thresholds for serum folate insufficiency (15.9 nmol/L) and erythrocyte folate insufficiency (906 nmol/L). Thus, low levels of folate or erythrocyte folate may contribute to the development of depression. Notably, the U-shaped relationship observed in Figure 6 suggests that high erythrocyte folate levels may also be associated with depression. This could be explained by the fact that erythrocyte folate concentration is commonly used as a primary indicator of folate sufficiency, and excessive folate supplementation has been linked to neurotoxicity. For instance, some studies have suggested that excessive or unmetabolized synthetic folate supplementation may have a negative impact on genetic programming and neuronal development (44, 45).

Therefore, in the chronic management of people with depression or depressive symptoms, attention should be paid to their dietary habits and nutritional status. When folate or erythrocyte folate levels fall below a certain threshold, supplementation should be considered. Depression can be managed by different therapeutic approaches including lifestyle changes, pharmacological interventions, and psychotherapeutic approaches (32), but combined therapeutic approaches have now been shown to be more effective (46). Altaf et al. (47) concluded that folate-assisted antidepressant medication improved depression scale scores, patient response, and remission rates. In experimental studies in mice, the administration of 50 mg/kg of folate daily prevented stress-induced oxidative damage and depressive behavior (48). A meta-analysis of randomized controlled trials found that adjuvant folate was significantly better than placebo in improving depressive symptoms for MDD (49). Finally, in conjunction with our findings, it is recommended that patients with moderate or higher levels of depression or depressed people who already have unhealthy eating habits may be given an appropriate intake of folic acid. From a clinical perspective, assessing folate levels in patients with depression can help identify those at risk of deficiency and provide some basis for considering folate supplementation as an adjunctive therapy for depression. For patients with low serum folate levels, folate supplementation may help improve treatment outcomes, but the dosage should be tailored to individual needs and clinical circumstances. Furthermore, clinicians should be aware of potential interactions between folate supplements and other medications that may affect folate metabolism. While further research is needed to establish optimal supplementation dosages, the findings of this study emphasize the potential benefits of folate supplementation in the treatment of depression, which could enhance treatment outcomes and improve patient care quality.

Our study has several limitations. First, we were unable to explore the folate levels of patients before their accompanying depression in this study, considering the information limitations of the database, which means we cannot establish a clear temporal relationship between depression and folate insufficiency. Second, this study evaluated the association between depressive status and folate levels, but the results cannot be extrapolated to cause and effect. Lower folate levels observed in depression may be secondary to poor dietary habits. Finally, our study was not conducted in a clinical setting, which limits the generalizability of our findings to real-world clinical practice. To mitigate these limitations, we suggest that in the future, studies should aim to include longitudinal data to explore the temporal relationship between depression and folate levels, and randomized controlled trials should investigate the potential benefits of folate supplementation in depressed individuals. The clinical trial assessing the effectiveness of folate supplementation in improving depression outcomes could provide more concrete evidence to guide treatment strategies.

## 5 Conclusion

Depression is associated with folate levels. The risk of serum folate insufficiency or erythrocyte folate insufficiency is higher after a positive depression. For different degrees of depressive symptoms, serum folate levels were significantly lower than normal in patients with moderate depression, while erythrocyte folate levels were lower than normal in patients with major depression. Therefore, attention should be paid to the dietary habits and nutritional status of patients with depression or depressive symptoms when they are undergoing long-term antidepressant treatment. Folic acid supplementation is recommended for patients with moderate or severe depression or for depressed patients who have developed unhealthy eating habits.

## Data Availability

The datasets presented in this study can be found in online repositories. The names of the repository/repositories and accession number(s) can be found at: https://wwwn.cdc.gov/nchs/nhanes/Default.aspx. The National Health and Nutrition Examination Survey (NHANES).
